# The knowledge, attitude, and practice toward fall prevention of parturients in the obstetric inpatient wards: a comparative cross-sectional study between nurses and parturients

**DOI:** 10.3389/fpubh.2025.1412111

**Published:** 2025-01-30

**Authors:** Jing Wen, Jiao Wen, Wenjuan Jing, Hongmei He, Jianhua Ren

**Affiliations:** ^1^Department of Obstetrics Nursing, West China Second University Hospital, Sichuan University, Chengdu, Sichuan, China; ^2^Key Laboratory of Birth Defects and Related Diseases of Women and Children (Sichuan University), Ministry of Education, Chengdu, Sichuan, China

**Keywords:** fall prevention, parturients, knowledge, attitude, practice

## Abstract

**Objective:**

This study aimed to compare the knowledge, attitudes, and practices of parturients and nurses regarding fall prevention among parturients, with the goal of understanding the cognitive factors contributing to post-delivery fall.

**Methods:**

A cross-sectional survey was conducted among obstetric nurses and women in obstetric ward within 2 days after vaginal delivery. The survey instrument included a self-design questionnaire comprising a demographic information sheet and a KAP questionnaire, which demonstrated acceptable reliability and validity.

**Results:**

A total of 705 nurses and 1,220 parturients completed the survey. Over half of both nurses and parturients exhibited reasonably good knowledge, positive attitudes and appropriate practices related to fall prevention. Significant associations were identified between the education level of parturients and their knowledge and attitudes. Nurses scored lower in the attitude dimension compared to parturients (4.32 ± 1.01 vs. 4.84 ± 0.36, *P* < 0.001), whereas nurses scored higher than parturients in the practice dimension (3.76 ± 0.44 vs. 3.57 ± 0.48, *P* < 0.001).

**Conclusion:**

Nurses should focus on enhancing the fall practices of parturients by emphasizing key points for fall prevention and providing guidance in situations of improper behaviors (e.g., unaccompanied activities). Further education programs for nurses should prioritize increasing their confidence and proficiency in fall prevention.

## 1 Introduction

Inpatient fall prevention is a critical topic in healthcare and is considered the index of medical care quality. Numerous studies have explored fall prevention in the medical field, while few have focused on postpartum falls although they are at the highest risk of falls among all patients (1). Delivery elements like blood loss during delivery, fatigue, hypotension, weakness, and pain medication leading to sedation or muscle relaxation, contribute to an increased risk of physical imbalance and falls (2). Hospital falls can lead to physical injuries, prolonged hospital stays, increased medical costs and disputes (3, 4). Substantial evidence supports the effectiveness of patient education programs in reducing fall rates (5). However, nurse-developed intervention often lack customization, neglecting aspects like risk attitude, independence, and advice receptiveness (5). The Knowledge-Attitude-Practice Model (KAP Model) posits that correct understanding of fundamental knowledge is prerequisite for individuals to develop beliefs and modify behaviors (6). The Registered Nurses’ Association of Ontario (RN) clinical practice guidelines for Preventing Falls and Reducing Injuries from Falls recommend assessing patients’ knowledge and fall-risk cognitive, as well as their prevention participation willingness (7). Tailor-made fall prevention strategies need to be implemented according to the characteristics of specific populations. In addition, evidence indicates that nurses’ favorable attitudes and knowledge regarding fall prevention education exert an impact on the occurrence of falls (8). However, extant studies have yet to conduct a specific examination of the knowledge, attitudes, and practices in relation to falls among parturients, nor have they delved into the disparities between the perspectives of nurses and those of patients. Consequently, the targets for intervention remain indeterminate.

We surveyed nurses and parturients about their fall prevention knowledge, attitudes, and practices. Understanding the gap in fall-related attitudes between them gives nurses the power to create precise methods and conduct real-time assessment, leading to enhanced patient safety.

## 2 Methods

### 2.1 Ethical considerations

All participants provided informed consent. Ethics committee approval was obtained from the West China Second University Hospital, Sichuan University Ethics Committee (decision no: 2021–217).

### 2.2 Design and participants

#### 2.2.1 Design

This study employed a descriptive cross-sectional survey design, utilizing self-report questionnaires to assess the knowledge, attitudes and practices related to hospital fall prevention among nurses and parturients.

The questionnaires were administered through an online survey platform called Wenjuan Xing, which offers functionality similar to Amazon Mechanical Turk. For data collection, Quick Response Codes were generated for both parturients and nurses to scan, enabling them to complete respective questionnaires. Researchers and assistants were present to provide assistance and answer any survey-related questions whenever they needed. It is important to note that all responses were kept anonymous.

#### 2.2.2 Participants

Parturients who gave birth to live infants between June 2021 and January 2022 at the obstetric department of a large maternity hospital in China were recruited. Inclusion criteria for parturients were as follows: (1) age ≥ 18 years; (2) delivery via vaginal birth; (3) ability to communicate and read in Chinese; (4) ability to provide informed consent. Exclusion criteria for parturients are as follows: (1) Having mental health disorders that significantly interfere with participation; (2) Having cognitive impairments or reading disorders; (3) Having severe physical illnesses; (4) Inability to provide informed consent due to any reason.

Nurses from obstetrics departments in 11 hospitals in Sichuan Province of China were recruited through convenience sampling. Inclusion criteria for nurses consisted of being active-duty hospital staff, while nursing interns and visiting nurses were excluded.

### 2.3 Measurement instrument

The survey comprised two questionnaires, one tailored for parturients and the other for nurses. Both questionnaires included sections for collecting demographic data and conducting a Knowledge, Attitudes, and Practices (KAP) survey.

The demographic data sheet for parturients included the following information: age, educational level, employment status, residence, monthly income, fall history during pregnancy, and fall history in the hospital. For nurses, the demographic data contained age, educational level, professional title, years of work experience, hospital level of employment, frequency of fall education received, experience with patient falls within the past 2 years.

The KAP questionnaires were designed in accordance with the KAP model. An expert panel consisting of five experts in obstetrical nursing reviewed the questionnaire and evaluated the representativeness of the items regarding the KAP scale. For the parturients’ questionnaire, the Item-Content Validity Index (I-CVI) ranged from 0.803 to 0.836, with a Scale-Content Validity Index for Universal Agreement (S-CVI/UA) of 0.831 and a Scale-Content Validity Index for Average (S-CVI/Ave) of 0.938. Similarly, for the nurses’ questionnaire, the I-CVI ranged from 0.793 to 0.814, with an S-CVI/UA of 0.891, and an S-CVI/Ave of 0.915. All of these values fell within acceptable ranges, indicating good content validity for the questionnaire. To ensure clarity and reliability, the questionnaire was piloted-tested with a sample of 50 nurses and 45 parturients to ascertain the intelligibility and reliability. The Cronbach’s *α* coefficient for the parturients’ questionnaire was 0.926, indicating excellent internal consistency. For the nurses’ questionnaire, the Cronbach’s *α* coefficient was 0.843, suggesting good internal consistency. While the questionnaires for both parturients and nurses shared similar dimensions, they varied in presentation due to the different roles and identities of the respondents, which were elaborated in the following sections: Knowledge Section, Attitude Section, Practice Section for Parturients and Nurses.

The knowledge section was designed to assess the identification of risk factors for falls, comprising 9 internal items and 3 external items. The questions were objective in nature with ‘correct’ or ‘incorrect’ options. Each correct answer received a score of 1, resulting in a total score ranging from 0 to 12. Participants’ overall knowledge was categorized by using a modified Bloom’s cut-off point: “good” if the score was over 80% of the total (10–12 points in this study), “moderate” if the score fell within 50–79% (6–9 points), and “poor” if the score was less than 50% (< 6 points). The attitude section was composed of 5 items, categorized into two dimensions. The first dimension contains 2 items focused on the parturients’ and nurses’ views of hospital falls, while the second dimension consisted of 3 items assessing attitudes toward fall prevention measures in the hospital. Response options ranged from 1 (strongly disagree) to 5 (strongly agree), resulting in the total score ranging from 5 to 25. According to the modified Bloom’s cut off, scores were categorized as “negative” with scores of 5–12, “neutral” with scores of 13–19, and “positive” with scores of 20–25. In the practice section for parturients, the parturients’ compliance with fall prevention measures was assessed through 7 questions. Among them, 5 items evaluated individual fall prevention behaviors, while 2 items examined communication practices between parturients and nurses. For nurses, the practice section consisted of 7 questions designed to evaluate the implementation of the fall prevention program. Among these, 5 items assessed the frequency of supervision practices by nurses regarding individual fall prevention behavior of parturients, while 2 items examined communication practices with parturients. The difference in the questionnaires between parturients and nurses was primarily related to the presentation of items. For example, in the parturients’ questionnaire, an item asked, “How often do you slowly change position or stand up?” While in the nurse’s questionnaire, the same concept was presented as “How often do you urge the parturients to slowly change position or stand up.” Responses for both groups ranged from 1 (never) to 4 (always), resulting in total scores ranging from 7 to 28. According to the modified Bloom’s cut off, scores were categorized as “negative” with scores of 7–13, “neutral” with scores of 14–22 and “positive” with score of 23–28.

### 2.4 Statistical analysis

For data analysis, we utilized SPSS IBM (version 22.0) software. Descriptive statistics, including frequency, percentage, mean, and standard deviation (SD), were computed for numerical data. The normality of the numerical distribution of all items was initially assessed using a 1-sample Kolmogorov–Smirnov (K–S) test. For groups of objects that followed a normal distribution, we employed independent sample t-test and analysis of variance (ANOVA) to compare their mean scores. In cases where indicators did not meet the criteria for normal distribution, non-parametric tests such as the Mann–Whitney *U* test and Kruskal–Wallis test were employed. Regression analysis was applied to determine independent factors associated with the levels of knowledge, attitude, and practice for both parturients and nurses. Statistical significance was established for all analyses with a *p*-value less than 0.05.

## 3 Results

### 3.1 Sample characteristics and univariate analysis of knowledge, attitude and practice among parturients and nurses

#### 3.1.1 Sample characteristics and univariate analysis of knowledge, attitude and practice among parturients

A total of 1,220 parturients participated in the survey. Among them, 84.3% were under the age of 35, and 89.8% had a fixed occupation. A small percentage (3.2%) reported a fall history during pregnancy, while 1.1% had experienced falls in the hospital setting. The demographic characteristics of parturients are detailed in Table 1.

**Table 1:** Univariate analysis of knowledge, attitude and practice against socio-demographic variables of parturients (*n* = 1,220).

Characteristic	*n* (%)	Parturients’ knowledge			Parturients’ attitude			Parturients’ practice		
		Mean ± SD	*Z*/*X*^2^	*P*	Mean ± SD	*Z*/*X*^2^	*P*	Mean ± SD	*Z*/*X*^2^	*P*
Age			−0.683	0.495		−1.464	0.143		−1.414	0.157
<35 years	1,029 (84.3)	11.33 ± 1.17			24.53 ± 1.25			25.25 ± 3.21		
≥35 years	191 (15.6)	11.36 ± 1.16			24.33 ± 1.55			25.74 ± 2.88		
Educational level			27.107	<0.001		23.072	<0.001		2.067	0.559
High school and below	61 (5.0)	10.51 ± 1.77			23.79 ± 1.94			25.21 ± 3.61		
College	213 (17.4)	11.22 ± 1.22			24.53 ± 1.21			25.33 ± 2.27		
Undergrad	674 (55.2)	11.37 ± 1.15			24.51 ± 1.29			25.43 ± 2.97		
Master’s and above	272 (22.3)	11.51 ± 0.91			24.50 ± 1.30			25.19 ± 3.15		
Employment			−2.544	0.011		−0.303	0.762		−0.379	0.704
Employed	1,100 (89.8)	11.45 ± 1.02			24.50 ± 1.31			25.28 ± 3.20		
Unemployed	120 (10.2)	10.98 ± 1.56			24.51 ± 1.22			25.05 ± 3.66		
Residence			−2.315	0.021		−3.401	0.001		−0.867	0.386
Urban	1,160 (95.1)	11.35 ± 1.13			24.54 ± 1.24			25.34 ± 3.13		
Country	60 (4.9)	10.88 ± 1.62			24.40 ± 1.50			25.00 ± 3.77		
Monthly income (yuan)			9.934	0.007		8.606	0.014		1.463	0.481
≤6,000	287 (23.5)	11.15 ± 1.33			24.33 ± 1.51			25.01 ± 3.51		
6,000–10,000	439 (36.0)	11.32 ± 1.23			24.50 ± 1.31			25.44 ± 2.93		
>10,000	494 (40.5)	11.43 ± 0.99			24.61 ± 1.14			25.41 ± 3.15		
Fall history during pregnancy			−0.074	0.941		−0.710	0.478		−0.977	0.328
Yes	39 (3.2)	11.00 ± 2.02			24.56 ± 1.37			25.56 ± 3.70		
No	1,181 (96.7)	11.34 ± 1.12			24.51 ± 1.28			25.32 ± 3.14		
Fall history in the hospital			−1.018	0.309		−1.638	0.101		−0.969	0.332
Yes	13 (1.1)	10.85 ± 2.48			25.00 ± 0.10			25.15 ± 2.48		
No	1,207 (98.9)	11.33 ± 1.15			24.51 ± 1.29			25.33 ± 3.17		

Univariate analysis was performed to compare the levels of knowledge, attitude and practice among parturients based on socio-demographic factors (Table 1). Statistically significant differences in knowledge levels were observed among groups with varying educational levels, employment status, residence and monthly incomes. Similarly, attitude levels showed statistically significant differences among different residence, education levels and monthly income brackets. However, there were no significant differences in practice levels.

#### 3.1.2 Sample characteristics and univariate analysis of knowledge, attitude and practice toward nurses

A total of 705 nurses participated in the survey. Among them, 67.9% were 25–34 years, and 68.1% held a bachelor’s degree and above. Approximately 52.6% of nurses reported participating in 3–5 fall prevention education sessions, while 10.4% had experience with patients’ falls in the hospital within the past 2 years (Table 2).

**Table 2:** Univariate analysis of knowledge, attitude and practice against socio-demographic variables of nurses (*n* = 705).

Characteristic	*n* (%)	Nurse’ knowledge			Nurse’ attitude			Nurse’ practice		
		Mean ± SD	*Z*/*X*^2^	*P*	Mean ± SD	*Z*/*X*^2^	*P*	Mean ± SD	*Z*/*X*^2^	*P*
Age (year)			3.688	0.297		7.977	0.046		2.171	0.538
<25	77 (10.9)	10.75 ± 0.73			22.62 ± 4.56			26.26 ± 3.46		
25–34	479 (67.9)	10.63 ± 1.10			21.98 ± 4.97			26.41 ± 3.09		
35–45	121 (17.2)	10.63 ± 0.73			20.95 ± 5.99			26.18 ± 2.81		
>45	28 (4.0)	10.64 ± 0.79			21.95 ± 5.11			26.14 ± 2.71		
Educational level			−0.027	0.978		−0.488	0.625		−0.790	0.430
College and below	225 (31.9)	11.56 ± 1.14			22.02 ± 4.72			26.27 ± 3.33		
Bachelor and above	480 (68.1)	11.49 ± 1.28			21.84 ± 5.22			26.37 ± 2.94		
Professional title			1.064	0.587		2.535	0.282		6.982	0.030
Nurse	150 (21.3)	11.59 ± 0.94			22.30 ± 4.54			26.37 ± 3.50		
Senior nurse	366 (51.9)	11.50 ± 1.33			21.93 ± 5.07			26.52 ± 2.82		
Supervisor nurse and above	189 (26.8)	11.49 ± 1.22			21.56 ± 5.46			26.01 ± 3.11		
Work experience			5.511	0.138		10.991	0.012		14.149	0.003
<3 years	115 (16.3)	11.65 ± 0.94			22.60 ± 4.56			26.71 ± 2.88		
3–5 years	157 (22.3)	11.50 ± 1.47			21.57 ± 5.19			26.02 ± 3.41		
5–8 years	127 (18.0)	11.62 ± 0.78			22.15 ± 5.27			26.97 ± 2.45		
≥8 years	306 (43.3)	11.43 ± 1.33			21.72 ± 5.12			26.13 ± 3.11		
Hospital level of employment			4.088	0.130		0.433	0.805		1.682	0.431
Class III Grade I	522 (74.0)	11.55 ± 1.19			21.89 ± 5.17			26.34 ± 3.06		
Class III Grade II	112 (15.9)	11.39 ± 1.53			22.33 ± 3.93			26.13 ± 3.37		
Class II Grade I	71 (10.1)	11.46 ± 0.92			21.38 ± 5.91			26.77 ± 2.41		
Number of fall prevention education session in the past 2 years			7.400	0.060		6.502	0.090		24.593	0.001
None	21 (3.0)	11.14 ± 1.68			22.29 ± 4.45			26.71 ± 2.49		
1–2	117 (16.6)	11.64 ± 1.17			22.53 ± 4.42			26.85 ± 3.12		
3–5	371 (52.6)	11.53 ± 0.96			21.56 ± 5.46			26.19 ± 3.07		
≥6	196 (27.8)	11.34 ± 1.81			21.91 ± 4.88			25.95 ± 2.90		
Experience of patient falling in the past 2 years			−1.314	0.189		−0.620	0.535		−2.128	0.033
No	632 (89.6)	11.55 ± 1.16			21.91 ± 5.10			26.43 ± 2.93		
Yes	73 (10.4)	11.22 ± 1.70			21.82 ± 4.98			25.59 ± 3.96		

Univariate analysis revealed statistical differences in the knowledge levels among groups based on patients’ fall experiences in the past 2 years. Significant differences in practice levels were observed among nurses with varying levels of work experience, professional title, the number of fall prevention education sessions attended in the past 2 years, and experiences with patients’ falls in the same time frame. However, there were no significant differences in attitude levels (Table 2).

### 3.2 Linear regression analysis of knowledge, attitude, and practice levels among parturients and nurses

The linear regression analyses illustrated that education level significantly influenced both fall knowledge and attitude among parturients (Table 3). Parturients with college, undergraduate and master’s degree and above scored higher in both knowledge and attitude compared to those with high school education and below (Table 3).

**Table 3:** Linear regression of knowledge, attitudes, and practices among nurse and parturients.

Variables	*B*	SB	*B*′	*T*	*P*
Parturients’ knowledge					
Residence_country	−0.063	0.038	−0.050	−1.640	0.101
(Reference: Residence_urban)					
Educational level_College	0.471	0.132	0.200	3.568	<0.001
Educational level_Undergrad	0.573	0.126	0.319	4.536	<0.001
Educational level_Master’s and above	0.654	0.135	0.304	4.834	<0.001
(Reference: Educational level_High school and below)					
Employment	0.115	0.090	0.038	1.285	0.199
(reference: unemployed)					
Monthly income _6,000–10,000 yuan	0.073	0.069	0.039	1.071	0.284
Monthly income _>10,000 yuan	0.133	0.069	0.073	1.930	0.054
(Reference: Monthly income <6,000 yuan)					
Parturients’ attitude					
Educational level_College	0.669	0.190	0.196	3.518	<0.001
Educational level_Undergrad	0.634	0.178	0.242	3.564	<0.001
Educational level_Master’s and above	0.698	0.191	0.224	3.662	<0.001
(Reference:Educational level_High school and below)					
Monthly income (yuan)_6,000–10,000	0.094	0.100	0.035	0.939	0.348
Monthly income (yuan)_>10,000	0.191	0.100	0.072	1.903	0.057
(Reference: Monthly income <6,000 yuan)					
Residence_country	−0.179	0.102	−0.050	−1.757	0.079
(Reference: Residence_urban)					
Nurses’ attitude					
Work experience_3–5 years	−0.832	0.668	−0.068	−1.245	0.214
Work experience_5–8 years	−0.267	0.717	−0.020	−0.372	0.710
Work experience_>8 years	−0.311	0.653	−0.030	−0.476	0.634
(Reference: work experience <3 years)					
Age (year)_25–34	−0.360	0.582	−0.034	−0.618	0.537
Age (year)_35–45	−1.482	0.790	−0.105	−1.875	0.061
Age (year)_>45	−0.467	1.244	−0.016	−0.376	0.707
(Reference: Age (year)_<25)					
Nurses’ practice					
Work experience_3–5 years	−0.873	0.457	−0.119	−1.911	0.056
Work experience_5–8 years	−0.003	0.505	0.001	−0.006	0.995
Work experience_>8 years	−0.643	0.495	−0.104	−1.301	0.194
(Reference: work experience <3 years)					
Number of fall prevention education session in the past two years_1–2	0.062	0.696	0.009	0.089	0.929
Number of fall prevention education session in the past two years_3–5	−0.537	0.681	−0.088	−0.787	0.431
Number of fall prevention education session in the past two years_6	−0.731	0.721	−0.089	−1.014	0.311
(Reference: Number of fall prevention education session in the past two years <1)					
Experience of patient falling in the past two years	−0.688	0.377	−0.069	−1.826	0.068
(Reference: no)					
Professional title_Senior nurse	0.526	0.404	0.086	1.304	0.193
Professional title_Supervisor nurse and above	0.240	0.495	0.035	0.484	0.629
(reference: Professional title_Nurse)					

### 3.3 Scores of knowledge, attitude and practice regarding fall prevention

Based on the modified Bloom’s cut-off point, the distribution of participants across different levels is shown in Table 4. A substantial majority of both nurses and parturients exhibited a good level of knowledge (93% vs. 90.8%). Similarly, a significant portion of both groups displayed a positive attitude toward fall prevention (66.1% vs. 91.2%). Furthermore, a substantial percentage of nurses and parturients showed appropriate practice levels (84.7% vs. 89.4%).

**Table 4:** The knowledge, attitude and practice levels of fall prevention respondents among nurses and parturients in obstetric inpatient wards.

	Nurses (*n* = 705) *N* (%)	Parturients (*n* = 1,220) *N* (%)
Knowledge		
<6 (poor)	5 (0.7)	3 (0.2)
6–9 (moderate)	44 (6.3)	109 (8.9)
10–12 (good)	656 (93.0)	1,108 (90.8)
Attitude		
5–12 (negative)	60 (8.5)	3 (0.2)
13–19 (neutral)	179 (25.4)	104 (8.5)
20–25 (positive)	466 (66.1)	1,113 (91.2)
Practice		
7–13 (negative)	3 (0.6)	3 (0.2)
14–22 (neutral)	104 (14.8)	126 (10.3)
23–28 (positive)	598 (84.7)	1,091 (89.4)

### 3.4 Comparison of knowledge, attitude and practice scores between respondents

Statistically significant differences were observed in attitude and practice between nurses and parturients (Table 5). Nurses scored significantly lower in attitude compared to parturients (4.32 ± 1.01 vs. 4.84 ± 0.36, *P* < 0.001). Conversely, nurse scored significantly higher in the practice dimensions compared to parturients (3.76 ± 0.44 vs. 3.57 ± 0.48, *P* < 0.001).

**Table 5:** Comparison of knowledge, attitude and practice scores between respondents.

		Nurses (*n* = 705) Mean ± SD	Parturients (*n* = 1,220) Mean ± SD	*Z*	*P*
Knowledge		0.96 ± 0.10	0.95 ± 0.10	1.523	0.128
Intrinsic factors	Dizzy	0.99 ± 0.09	0.98 ± 0.12	1.612	0.107
	Weakness	0.99 ± 0.08	1.00 ± 0.06	−1.131	0.258
	Hunger	0.95 ± 0.21	0.97 ± 0.18	−1.161	0.246
	Sedatives/hypnotics	0.98 ± 0.15	0.85 ± 0.36	8.984	<0.001
	Chronic illness	0.97 ± 0.12	0.95 ± 0.18	1.101	0.283
	Improper footwear	0.96 ± 0.13	0.96 ± 0.18	−0.049	0.961
	Rapidly changing position or standing up	0.97 ± 0.16	0.97 ± 0.16	0.195	0.846
	Fall history in 6 months	0.88 ± 0.33	0.74 ± 0.44	7.038	<0.001
	Inadequate exercise	0.95 ± 0.19	0.93 ± 0.15	1.152	0.262
Extrinsic factors	Slippery surfaces	0.99 ± 0.08	0.99 ± 0.12	0.760	0.447
	Poor lighting	0.99 ± 0.11	0.98 ± 0.13	1.158	0.247
	Height of bed or chairs	0.97 ± 0.17	0.97 ± 0.17	−0.143	0.886
Attitude		4.32 ± 1.01	4.84 ± 0.36	−16.855	<0.001
Views on falls Views on fall prevention measures	Falls are preventable	3.98 ± 1.01	4.73 ± 0.47	−24.562	<0.001
	Falls can cause serious injuries	4.23 ± 1.05	4.83 ± 0.47	−17.850	<0.001
	Fall prevention education can prevent falls	4.43 ± 1.14	4.80 ± 0.37	−13.577	<0.001
	Individual assessment of fall risk can prevent falls	4.33 ± 1.08	4.76 ± 0.56	−11.435	<0.001
	Prepared assistant facilities can prevent falls	4.44 ± 1.14	4.91 ± 0.29	−12.074	<0.001
Practice		3.76 ± 0.44	3.57 ± 0.48	8.637	<0.001
Individual practice	Urge/slowly changing position or standing up	3.79 ± 0.48	3.27 ± 0.43	12.384	<0.001
	Urge/proper footwear	3.75 ± 0.51	3.80 ± 0.55	−4.136	<0.001
	Urge/correctly using bedrail	3.79 ± 0.47	3.74 ± 0.52	−2.267	0.023
	Urge/keep proper height of bed or chairs	3.75 ± 0.32	3.66 ± 0.45	1.356	0.201
	Urge/keep bright field when moving	3.77 ± 0.48	3.59 ± 0.42	4.869	<0.001
Communication practice	Told/asking for help in unaccompanied activities	3.82 ± 0.45	3.22 ± 0.61	13.104	<0.001
	Fall education/educated before initial early ambulation	3.74 ± 0.36	3.68 ± 0.36	1.023	0.344

## 4 Discussion

### 4.1 Actors associated with knowledge, attitude, and practice levels regarding hospital falls among parturients and nurses

The survey findings demonstrated that most parturients and nurses had a good knowledge, attitude and practice level in postpartum fall. This good level could be attributed to facility initiatives implemented during the study period, which included discussion of fall risk factors with parturients.

The results highlight that the education level significantly affects parturients’ knowledge and attitude toward fall prevention. This finding consistent with previous study reporting that individuals with higher education tend to be more attentive to safety issues (9). Therefore, health education initiatives targeted fall prevention should prioritize lower-educated parturients.

### 4.2 The disparities between nurses and parturients within the dimension of fall prevention knowledge

There was no significant differences in the total knowledge score between nurses and parturients. However, it is noteworthy that in terms of identifying “Fall history in 6 months” and “sedative-hypnotic drugs” as factors contributing to falls, parturients scored significantly lower than nurses. This disparity could potentially be attributed to the difference in professional knowledge and training. Nurses, with their extensive medical education and clinical experience, are more likely to be aware of the various risk factors associated with falls (10). Parturients usually have limited access to such detailed medical knowledge. As observed in previous study, parturients attributed their falls to insufficient comprehension of epidural analgesia’s effects and duration (2). Healthcare providers could create specific educational materials and programs for parturients, such as brochures, videos, or counseling sessions, covering common and pregnancy/postpartum-specific fall risks. For nurses, it accentuates the importance of strengthening communication with parturients post-medication administration to guarantee their awareness of the attendant risks. Moreover, particular attention should be directed toward patients with a history of falls, as they are more vulnerable and require more intensive care and education to further mitigate the potential for recurrent falls and associated complications (11).

### 4.3 The disparities between nurses and parturients within the dimension of fall prevention attitude

This study showed that nurses had a significantly lower attitude score toward fall prevention than parturients. Furthermore, nurses scored the lowest on the item of “Falls are preventable.” This unexpected finding may be attributed to several factors. Firstly, nurses, due to their extensive exposure to various patient cases and clinical scenarios, might have developed a sense of resignation or pessimism regarding the complete prevention of falls. Nurses might have a better understanding of the complex and unpredictable nature of factors contributing to falls, such as complex patient conditions, insufficient staffing, ineffective staff communication, and deficiencies in safety-related knowledge, skills, and attitudes (12, 13). Many nurses reported being unable to prevent falls despite perceived fall-prevention knowledge; they identified fall incidents due to patients’ non-cooperation, despite several fall-prevention educations, and only 46% believed falls were preventable (14, 15). Secondly, the communication and feedback mechanisms within the healthcare system may contribute to this phenomenon. Insufficient recognition or positive reinforcement for nurses’ endeavors in fall prevention, along with the harsh penalties for patient falls may result in nursing staff experiencing anxiety, which not only impairs their capacity to deliver safe care with assurance but also fosters a sense of disempowerment in their role of ensuring safety (12). In contrast, due to the concern about the injuries that falls may cause, patients may have a more positive attitude toward fall prevention (16). Additionally, they might hold a more rudimentary perception, presuming basic safety guidelines suffice for fall prevention (17). This divergence in perception could underpin the attitudinal disparity.

To rectify this divergence and enhance overall fall prevention, it is imperative to target these latent issues. For parturients, hospitals should capitalize on parturients’ high awareness, involving them in fall-prevention activities. For nurses, healthcare institutions ought to provide greater support and allocate more resources, enhance communication channels, and review incentive mechanisms to inspire and encourage nurses. Additionally, future research could explore deeper into factors influencing nurses’ attitudes and develop better interventions.

### 4.4 The disparities between nurses and parturients within the dimension of fall prevention practice

In our study, parturients had a significantly more positive attitude toward fall prevention than nurses; nevertheless, parturients scored significantly lower in fall prevention practice compared to nurses. This interesting paradox may be attributed to several factors. Firstly, despite a positive attitude, parturients may lack an in-depth understanding of the actual fall risks related to their ability to mobilize. For some parturients, a lack of awareness of their leg strength being insufficient for walking was a contributing factor (2, 18). Secondly, the training and experience that nurses receive equips them with a sense of duty and routine in implementing fall prevention measures, regardless of their personal attitudes (14). Their education provides them with the requisite knowledge and skills to identify fall risks and take appropriate action, and they are accustomed to following established protocols in the clinical setting. This adherence to procedure may not necessarily be accompanied by a high level of personal enthusiasm or positive attitude, but it contributes to effective practice nonetheless. To bridge the gap between parturients’ attitude and practice, one efficacious approach would be to augment the fall prevention education furnished to parturients during the antenatal and postpartum periods. Parturients need targeted education to understand postnatal changes. The development of individualized fall prevention strategies which take into account each parturient’s physical and psychological state is necessary. Nurses could also be trained in more effective communication strategies to better engage parturients.

Additionally, parturients had the lowest scores in help-seeking practice compared to nurses. One possible reason accounting for this phenomenon could be that patients are reluctant to interrupt or burden the staff (18). Another possible reason undermining the partnership between nurse and patient might be the delay in providing mobility assistance (19). To address the extant issue, hospitals are obliged to establish lucid communication channels and well-defined protocols, thereby facilitating parturients in expressing their needs expeditiously and without inhibition. Simultaneously, hospitals should manage human resources well, improve nursing services, and reduce assistance delays.

## 5 Limitation

First, the use of convenience sampling may limit the representativeness of the respondents and the generalizability of the study’s findings. Second, data collected from nurses and parturients from different hospitals hindered the exploration of causality between nurses’ characteristics and patients’ fall behavior.

## 6 Conclusion

The overall knowledge, attitude, and practice about hospital falls among parturients and nurses was positive and appropriate. While overall knowledge levels were comparable, specific deficiencies in knowledge were identified among parturients in relation to certain fall risk factors. With regard to practice, a paradoxical situation was observed, whereby parturients displayed a more positive attitude but exhibited poorer practice. These findings highlight the need for targeted educational interventions. For parturients, educational programs should not only concentrate on increasing their awareness of specific fall risk factors, but also on enabling them to translate their positive attitude into effective preventive actions. It is the obligation of medical personnel to facilitate patient involvement in the treatment process and practice patient-centered care. For nurses, continuous professional development in fall prevention should be emphasized to ensure they can provide accurate and comprehensive guidance to parturients. Hospitals should provide adequate support and incentives for nurses, facilitate clear and transparent communication, effectively manage resources, and address delays in assistance to ensure optimal patient care. Future research should dig into nurse attitude factors and create better interventions.

## Data Availability

The original contributions presented in the study are included in the article/supplementary material, further inquiries can be directed to the corresponding author.

## References

[1] Chen KH Chen LR Su S. Applying root cause analysis to improve patient safety: decreasing falls in postpartum women. Quality & safety in health care, (2010) 19:138–43. doi: 10.1136/qshc.2008.028787 20351162

[2] Lockwood S Anderson K. Postpartum safety: a patient-centered approach to fall prevention. MCN Am J Matern Child Nurs. (2013) 38:15–8. doi: 10.1097/NMC.0b013e31826bae4b 23060327

[3] Liu J Wong ZSY So HY. Automatic patient fall outcome extraction using narrative incident reports. Stud Health Technol Inform. (2022) 290:724–8. doi: 10.3233/SHTI220173, PMID: 35673112

[4] Dykes PC Curtin-Bowen M Lipsitz S Franz C Adelman J Adkison L. Cost of inpatient falls and cost-benefit analysis of implementation of an evidence-based fall prevention program. JAMA Health Forum. (2023) 4:e225125. doi: 10.1001/jamahealthforum.2022.5125, PMID: 36662505 PMC9860521

[5] Heng H Jazayeri D Shaw L Kiegaldie D Hill AM Morris ME. Hospital falls prevention with patient education: a scoping review. BMC Geriatr. (2020) 20:140. doi: 10.1186/s12877-020-01515-w, PMID: 32293298 PMC7161005

[6] Glanz K Rimer BK Viswanath K. Health behavior and health education: theory, research, and practice. San Francisco, CA: John Wiley & Sons (2008).

[7] Registered Nurses’ Association of Ontario. Preventing falls and reducing injury from falls. 4th ed. Toronto, ON: Registered Nurses’ Association of Ontario (2017).

[8] Cho MY Jang SJ. Nurses’ knowledge, attitude, and fall prevention practices at south Korean hospitals: a cross-sectional survey. BMC Nurs. (2020) 19:108. doi: 10.1186/s12912-020-00507-w, PMID: 33292192 PMC7686720

[9] Gamage N Rathnayake N Alwis G. Knowledge and perception of falls among community dwelling elderly: a study from Southern Sri Lanka. Curr Gerontol Geriatr Res. (2018) 2018:7653469. doi: 10.1155/2018/7653469, PMID: 30002676 PMC5996422

[10] Takase M Kisanuki N Nakayoshi Y Uemura C Sato Y Yamamoto M. Exploring nurses' clinical judgment concerning the relative importance of fall risk factors: a mixed method approach using the Q methodology. Int J Nurs Stud. (2024) 153:104720. doi: 10.1016/j.ijnurstu.2024.104720, PMID: 38408403

[11] Schoberer D Breimaier HE Zuschnegg J Findling T Schaffer S Archan T. Fall prevention in hospitals and nursing homes: clinical practice guideline. Worldviews Evid-Based Nurs. (2022) 19:86–93. doi: 10.1111/wvn.12571 35262271 PMC9310602

[12] Garcia A Bjarnadottir RRI Keenan GM Macieira TGR. Nurses' perceptions of recommended fall prevention strategies: a rapid review. J Nurs Care Qual. (2022) 37:249–56. doi: 10.1097/NCQ.0000000000000605, PMID: 34775419 PMC9095763

[13] Fehlberg EA Cook CL Bjarnadottir RI McDaniel AM Shorr RI Lucero RJ. Fall prevention decision making of acute care registered nurses. J Nurs Adm. (2020) 50:442–8. doi: 10.1097/NNA.0000000000000914, PMID: 32826513 PMC7592292

[14] Ayton DR Barker AL Morello RT Brand CA Talevski J Landgren FS. Barriers and enablers to the implementation of the 6-PACK falls prevention program: a pre-implementation study in hospitals participating in a cluster randomised controlled trial. PLoS One. (2017) 12:e0171932. doi: 10.1371/journal.pone.0171932, PMID: 28207841 PMC5313144

[15] Jang SG Ock M Kim S. Qualitative comparison of perceptions regarding patient engagement for patient safety by physicians, nurses, and patients. Patient Prefer Adherence. (2024) 18:1065–75. doi: 10.2147/PPA.S456050, PMID: 38854478 PMC11162203

[16] Shuman C Liu J Montie M Galinato JG Todd MA Hegstad M. Patient perceptions and experiences with falls during hospitalization and after discharge. Appl Nurs Res. (2016) 31:79–85. doi: 10.1016/j.apnr.2016.01.009, PMID: 27397823

[17] Kiyoshi-Teo H McMahon SK Northup-Snyder K Cohen DJ. Older People's descriptions of their engagement in fall prevention. West J Nurs Res. (2024) 46:10–8. doi: 10.1177/01939459231211803, PMID: 37950361 PMC13370804

[18] Heng H Slade SC Jazayeri D Jones C Hill AM Kiegaldie D. Patient perspectives on hospital falls prevention education. Front Public Health. (2021) 9:592440. doi: 10.3389/fpubh.2021.592440, PMID: 33796493 PMC8007862

[19] Radecki B Reynolds S Kara A. Inpatient fall prevention from the patient’s perspective: a qualitative study. Appl Nurs Res. (2018) 43:114–9. doi: 10.1016/j.apnr.2018.08.001, PMID: 30220357

